# Comparative Analysis of the Oxygen Supply and Viability of Human Osteoblasts in Three-Dimensional Titanium Scaffolds Produced by Laser-Beam or Electron-Beam Melting

**DOI:** 10.3390/ma6115398

**Published:** 2013-11-21

**Authors:** Anika Jonitz-Heincke, Jan Wieding, Christoph Schulze, Doris Hansmann, Rainer Bader

**Affiliations:** 1 Biomechanics and Implant Technology Research Laboratory, Department of Orthopaedics, University Medicine Rostock, Doberaner Strasse 142, Rostock 18057, Germany; E-Mails: jan.wieding@med.uni-rostock.de (J.W.); christoph.schulze@med.uni-rostock.de (C.S.); doris.hansmann@med.uni-rostock.de (D.H.); rainer.bader@med.uni-rostock.de (R.B.); 2 Department of Orthopaedic Surgery, Armed Forces Hospital Westerstede, Lange Str. 38, Westerstede 26655, Germany

**Keywords:** human osteoblasts, Ti6Al4V, laser-beam melting, electron-beam melting, scaffold, macropores

## Abstract

Synthetic materials for bone replacement must ensure a sufficient mechanical stability and an adequate cell proliferation within the structures. Hereby, titanium materials are suitable for producing patient-individual porous bone scaffolds by using generative techniques. In this *in vitro* study, the viability of human osteoblasts was investigated in porous 3D Ti6Al4V scaffolds, which were produced by electron-beam (EBM) or laser-beam melting (LBM). For each examination, two cylindrical scaffolds (30 mm × 10 mm in size, 700 µm × 700 µm macropores) were placed on each other and seeded with cells. The oxygen consumption and the acidification in the center of the structures were investigated by means of microsensors. Additionally, the synthesis of pro-collagen type 1 was analyzed. On the LBM titanium scaffolds, vital bone cells were detected in the center and in the periphery after 8 days of cultivation. In the EBM titanium constructs, however, vital cells were only visible in the center. During the cultivation period, the cells increasingly produced procollagen type 1 in both scaffolds. In comparison to the periphery, the oxygen content in the center of the scaffolds slightly decreased. Furthermore, a slight acidification of the medium was detectable. Compared to LBM, the EBM titanium scaffolds showed a less favorable behavior with regard to cell seeding.

## 1. Introduction

Segmental bone defects can be a result of fractures, traumas, tumors or endoprosthetic loosening. Currently, autologous and allogenic bone grafts are used for the treatment of these defects [1,2]. However, such grafts can be used to a very limited extent only, which is due to their limited availability, risks associated with extraction from the donor site, infections and the risk of immunological reactions to allogenic grafts [3,4]. Therefore, it is necessary to find alternatives in the form of synthetic, porous three-dimensional (3D) bone substitute materials that can be inserted into bone defects. For this purpose, the focus of research is on calcium phosphates as well as metals like titanium and its alloys. These materials can be used in clinical applications, are available in sufficient quantities and can be produced patient-individually by means of various manufacturing techniques (e.g., 3D printing, additive production methods). A lot of mechanical and biological conditions must be taken into account to ensure that the bone can grow into the synthetic materials. The different mechanical properties of bone substitute materials decide whether the materials are used in load-bearing or non-load-bearing areas, because these areas are subjected to different levels of mechanical stress. Mismatching between bone substitute material and the surrounding bone tissue can lead to a change in the mechanical load distribution within the tissue, as a result of which tissue growth into the material will be inhibited or the implant will loosen [5,6]. For this reason, the mechanical properties of the materials have been adapted to the biomechanical properties of the bone [7]. The mechanical compressive strength of scaffolds made of titanium alloys are comparable to that of human cortical bone [8].

Implants made of titanium are widely used in both orthopedic surgery and in the dental sector [9]. Porous structures are especially suitable for the management of large bone defects, since they show a high degree of mechanical stability [10]. Furthermore, good bone cell integration was already demonstrated for such structures *in vitro* [9,11,12]. The porosity of implants can either be provided by a foam-like structure with irregular pore size or by a lattice structure with regular pores. The latter are produced by additive manufacturing methods [8]. Porosity plays an important part in reducing stiffness mismatching between implants and the surrounding bone tissue. Furthermore, porosity, pore size and interconnected pores play an important biological role in ensuring bone ingrowth into the structures and hence in creating a lasting and stable bonding of the implant within the bone stock [13]. 

To ensure sufficient cell distribution, the structures of the 3D bone substitute materials have to offer an artificial surface on which the cells can migrate, proliferate and differentiate [14]. It should be kept in mind, however, that with increasing implant size gradients in cellular seeding and differentiation may occur between the internal and external structures [15,16,17]. This is mainly due to the fact that the cells in the interior are insufficiently supplied with nutrients and oxygen [17]. 

In living tissues, nutrients, oxygen and waste products are transported by the blood flow. Due to the proximity of the cells to a blood vessel, all cells are sufficiently supplied with nutrients [15]. However, the implantation of a bone substitute material leads to a temporary interruption of the blood flow, so that oxygen and nutrients have to be transported over several millimeters or centimeters by diffusion processes [15]. Since an adequate oxygen and nutrient supply to the cells is limited to a maximum of 200 µm *in vitro* [17], a higher porosity of the bone substitute materials should accelerate vascularization within the structures in order to ensure oxygen and nutrient supply as well as the removal of metabolic end products [18]. It takes several days to months for blood vessels to grow into cell-seeded scaffolds, so that an initial insufficient oxygen supply within the structures after implantation can be assumed [15,17]. Additionally, larger distances, in both native tissue and bone substitute materials, can cause imbalances between oxygen supply and oxygen consumption [19]. 

The objective of this *in vitro* study was to examine the oxygen supply and viability of human osteoblasts within 3D titanium scaffolds by using an established test setup [20]. For this purpose, scaffolds of the same size and porosity were produced by additive manufacturing processes using electron-beam (EBM) or laser-beam melting (LBM) techniques. Both titanium constructs were thus to be assessed for their biological suitability to draw conclusions about the different manufacturing processes and the design of pore size and pore arrangement with respect to bone cell viability and distribution.

## 2. Materials and Methods

### 2.1. Isolation and Cultivation of Human Primary Osteoblasts

Human primary osteoblasts were isolated and cultivated under standard conditions [20]. The cells were isolated under sterile conditions from femoral head spongiosa of patients who underwent implantation of total hip endoprosthesis. The femoral heads were made available after obtaining written consent of the patients and prior approval of the local ethics committee (registration number: A 2010-10). Bone cells of a total of 14 living donors (7 female, 62 ± 11 years; 7 male, 66 ± 9 years) were used for the *in vitro* tests.

Human osteoblasts in the third cell passage were seeded on the scaffolds (see Chapter 2.3). For this purpose, supernatant liquid of the culture medium was removed, the cells were rinsed with PBS (PAA, Coelbe, Germany) and subsequently detached from the bottom of the cell culture flask by means of trypsin/EDTA (Gibco^®^ Invitrogen, Darmstadt, Germany). After a centrifugation step, the cell pellet was resuspended in a defined medium volume and a cell count was performed using a Thoma counting chamber.

### 2.2. Titanium Scaffolds

**Figure 1:**
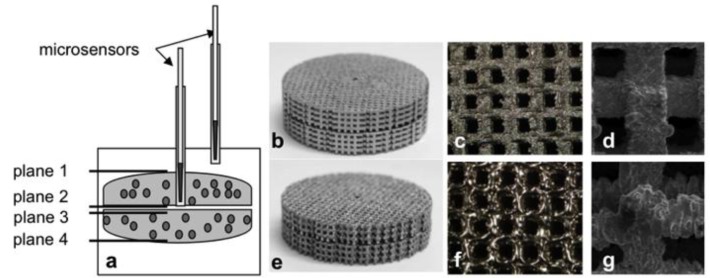
Presentation of the test setup with titanium scaffolds (**a**); (**b**–**d**) laser-beam melted titanium; (**e-g**) electron-beam melted titanium. Two scaffold discs were placed on each other (b, e), and each of these double constructs was then inserted into one well of a 6-well cell culture plate and seeded with cells. Pore arrangement in the titanium double modules (c, f). Scanning electron microscopy (SEM) of the two titanium surfaces (d, g).

The titanium scaffolds used in the tests were produced by additive manufacturing methods based on a CAD model and selective laser-beam melting (SLM Solutions GmbH, Lübeck, Germany). In addition, this study included the use of titanium scaffolds that were produced by selective electron-beam melting (Institute for Materials Science, University of Erlangen-Nuremberg, Erlangen, Germany). Titanium powder (Ti6Al4V) was used for both manufacturing processes [10]. As described by Koike *et al.* [21], the additive manufacturing of the constructs comprised three steps: distribution of the titanium powder, heating and melting by laser or electron beam. The steps were repeated until the constructs were completely in accordance with the specific CAD design [21]. The scaffolds were 5 mm in height and 30 mm in diameter and had a pore size of 700 × 700 µm in all three spatial directions (Figure 1c,d,f,g). 

### 2.3. Test Setup and Seeding of the Titanium Scaffolds

The test setup consisted of two scaffold discs which were placed on each other to form a 10-mm-high overall construct (Figure 1a,b,e). The lowest plane (plane 4) was in direct contact with the bottom of the cell culture plate. The upper disc had a central hole for inserting the microsensors.

Two scaffold discs were positioned over each other in one well of a 6-well culture plate, so that plane 1 could be seeded with cells. Prior to seeding, the titanium scaffolds were covered with complete medium to eliminate air bubbles. Subsequently, a total of 4 × 10^5^ cells were pipetted on the surface of plane 1 point by point in 10 µl drops. After an adherence period of 45 minutes, the cells were overlaid with complete medium containing osteogenic additives and then incubated under standard conditions for eight days. The cell culture medium was changed three times a week.

### 2.4. Viability Testing and Quantification of Procollagen Type 1

To analyze the viability of cells on the two bone substitute materials, a metabolic activity test (WST-1, Roche, Penzberg, Germany) and live/dead staining (LIVE/DEAD^®^ viability/cytotoxicity kit; Invitrogen) were performed. 

The WST-1 test is used to determine the mitochondrial dehydrogenase activity of cells. The cells turn over the tetrazolium salt WST-1 into formazan, which resulted in a color change. This change can subsequently be quantified in a microplate reader (Dynex Technologies, Denkendorf, Germany) at 450 nm (reference: 630 nm). To evaluate the metabolic activity of cells on the scaffolds, the WST-1 test was performed both after 24 hours (day 1) and at the end of the test (day 8). The overall construct was overlaid with a defined volume of the WST-1/medium reagent (ratio 1:10) and incubated at 37 °C and 5% CO_2_ for 60 min. A blank value was used in each test series. Subsequently, 200 µL aliquots were transferred into a 96-well cell culture plate for double measurement, and the absorption in the plate reader was determined.

The live/dead staining reagent contains the two fluorescence dyes calcein AM and ethidium homodimer 1. Calcein AM is a membrane-permeable acetoxymethyl ester of calcein, which is hydrolysed intracellularly to calcein by endogenous esterases. As calcein is membrane-impermeable, it will remain within the intact cells, which are therefore fluorescent green (ex/em 495/515 nm). Ethidium homodimer is a nuclear stain which emits red fluorescence after DNA binding (ex/em 495/635 nm). It enters cells through damaged membranes and can therefore be used for identifying dead cells. Both fluorescent dyes were dissolved in PBS according to the manufacturer’s instructions. Then, the scaffolds were incubated at room temperature in a darkened environment for 30 minutes. Unless stated otherwise, the cells were examined under a microscope with an objective lens with four-fold magnification. The respective images of live and dead cells were taken separately but in the same position, using a fluorescence microscope (Nikon ECLIPSE TS100, Nikon GmbH, Duesseldorf, Germany). Subsequently these images were superimposed by means of freely available image editing software (GIMP 2.6.6, GIMP-Team), so that a composite image of vital and dead cells was developed.

The synthesis of type 1 pro-collagen by human osteoblasts was determined using an enzyme-linked immunosorbent assay (ELISA) (C1CP; Quidel, Marburg, Germany). For this purpose, supernatant medium was collected during each medium change and then stored at −20 °C. The test was carried out according to the manufacturer’s instructions. To determine the protein quantity, standard curves with defined protein concentrations and a defined optical density were generated. The absorption of the samples was determined at 405 nm using a microplate reader (Dynex Technologies, Denkendorf, Germany).

### 2.5. Monitoring of Oxygen and pH Value

To measure the oxygen concentration and the pH value within the different titanium scaffolds, special microsensors were used (oxygen: Oxygen Micro-Optode, type PSt1; pH value: pH Microsensor; both manufactured by: Presens, Regensburg, Germany). These sensors consist of an optical fibre with a tip that is less than 150 µm in diameter. To protect the fragile sensors, they were sheathed in a hollow needle that was 0.4 mm in diameter. These hollow needles were placed in other hollow needles (1.02 mm in diameter) to increase their stability during measurements. Therefore, we could distinguish between two different regions: (a) the *center* within the scaffold (between plane 2 and 3); and (b) the *periphery* above plane 1 (Figure 1a).

Before each test series, the oxygen sensors were calibrated in oxygen-free water and in a water-saturated environment according to the manufacturer’s instructions. The pH sensors were also calibrated before each test series, using pH buffer solutions in ascending order from pH 4 to pH 7 (all manufactured by: Roth, Karlsruhe, Germany) according to the manufacturer’s instructions. 

Over a period of eight days, the oxygen content and acidification were measured daily for 30 min both in the center and on the periphery.

### 2.6. Statistical Evaluation

Human bone cells from 14 separate donors were used for the respective analyses. The data obtained were presented as mean values ± standard deviations. The statistical significance levels of the differences between mean values were calculated using a one-way ANOVA (post-hoc LSD). All statistical calculations were conducted using SPSS 15.0 for Windows (SPSS Inc., Chicago, IL, USA). The level of significance was *p* < 0.05.

## 3. Results 

### 3.1. Cell Viability and Collagen Synthesis 

The metabolic activity of cells was determined by a WST-1 assay on both titanium scaffolds after 24 hours and after eight days of cultivation. Compared to the metabolic activity after 24 hours, a decrease in metabolic activity by 37% was measured after eight days in the EBM titanium constructs, whereas metabolic activity in the LBM titanium constructs showed an insignificant increase by 92%. Hereby, at the end of cultivation, both scaffolds showed the same level of cell metabolic activity. 

**Figure 2:**
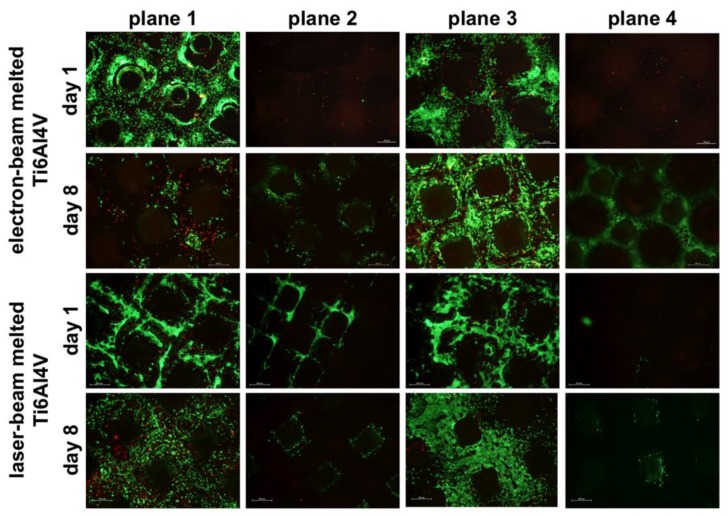
Exemplary illustration of the viability of human bone cells on the four planes of the titanium scaffolds on day 1 and day 8 (*n* = 3; green = living cells; red = dead cells; scale bar: 500 µm).

In addition to the cell viability tests, live/dead staining was performed at both time points. After 24 hours, a large number of vital cells were detected on planes 1 and 3 on both titanium scaffolds. Nevertheless, the cell distribution on both planes was inhomogeneous because of the pointwise cell seeding procedure. After eight days of incubation, plane 1 and 3 of the LBM titanium bodies were densely seeded with vital cells. Isolated dead cells were only identified on the first plane. In contrast, the EBM titanium showed only few spots with vital cells on plane 1 and many live cells on plane 3 after the end of the test. Additionally, on plane 3 a good cell distribution could be shown. However, a lot of dead cells were detected on both planes. After the end of testing period, on both titanium scaffolds, the second plane also showed initial seeding (Figure 2). 

For measuring the procollagen type 1 content during the cultivation period, the medium supernatant was collected on day 2, 4 and 7. For this purpose, the supernatants were removed with a standard syringe through the hollow needles of the center and the periphery and afterwards analyzed by ELISA. Both scaffolds showed an increase in procollagen type 1 levels in the course of cultivation, with higher levels being observed in the LBM titanium constructs (Table 1).

**Table 1:** Procollagen type 1 levels (in ng/mL) of human osteoblasts in electron-beam melting (EBM) and laser-beam melting (LBM) titanium scaffolds.

Time	Type of scaffold tested			
	EBM titanium		LBM titanium	
	Periphery	Center	Periphery	Center
Day 2	69.5 ± 40.1	88.5 ± 32.9	72.1 ± 26.8	64.1 ± 16.2
Day 4	107.7 ± 44.2	109.6 ± 48.4	236.6 ± 106.7	204.7 ± 88.0
Day 7	150.7 ± 78.5	214.7 ± 95.1	307.6 ± 115.8	328.5 ± 132.2

### 3.2. Oxygen Supply and Acidification in Titanium Scaffolds

**Figure 3:**
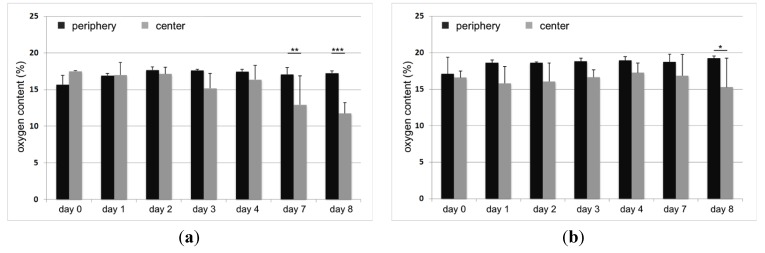
Oxygen concentration in a static 3D culture of human osteoblasts (*n* = 3). The measurements were performed in the center and on the periphery of EBM (left) and LBM (right) titanium scaffolds. The mean values ± standard deviations are presented. The statistical analysis was performed using an ONEWAY ANOVA (post-hoc LSD). The statistical significance is based on the oxygen concentration as determined by the respective peripheral measurement (*p < 0.05; **p < 0.01; ***p < 0.001).

In addition to the viability measurements, oxygen concentration and acidification were measured. The oxygen measurements in the titanium constructs showed slight differences in oxygen concentration from day 0 to day 4. At day 7, a significant difference (*p* = 0.002) between periphery and center was determined in the EBM titanium scaffold (Figure 3a). In the LBM titanium, a significant decrease (*p* = 0.013) was only detected on the eighth day (Figure 3b). Oxygen concentration in the center of the scaffolds decreased from 17.5% to 11.76% (EBM titanium) and from 16.56% to 15.26% (LBM titanium) (Figure 3). In the course of cultivation, both titanium constructs also showed a slight acidification both on the periphery and in the center.

## 4. Discussion

The use of synthetic materials is limited by the insufficient nutrient and oxygen supply to the cells seeding on such implants [17]. In particular with large bone substitute materials, oxygen is the limiting factor due to its low solubility and diffusion capacity in aqueous solution [15]. Since there is no vascularization from the outset, oxygen supply gradients between the inside and the outside develop after a short time, which can have far-reaching consequences for cell survival in the center. Therefore, it is necessary to analyze the oxygen partial pressures within the bone substitute materials, to draw conclusions with regard to optimize the pore design. In this study, an established test setup was used, which made it possible to examine different bone substitute materials in a static cell culture with regard to their seeding with human bone cells and the oxygen supply to these cells [20]. The bone substitute materials used had the same dimensions and were made of a titanium alloy by applying EBM and LBM techniques.

### 4.1. Influence of the Bone Substitute Materials on Cell Survival and Synthesis Capacity

Depending on the production methods, the titanium scaffolds showed different levels of cell survival. Initially, good cell seeding was demonstrated on both surfaces. In the course of cultivation, however, the EBM titanium scaffolds showed worse cell-seeding characteristics than LBM titanium. These results were confirmed by the metabolic activity tests. Although we could determine an initially higher metabolic activity of cells in the EBM scaffolds compared to the LBM ones, a clearly decrease after eight days was observed. In contrast, the metabolic activity in the LBM constructs increased. Therefore, in accordance with Hollander *et al.*, better biological compatibility was thus demonstrated for LBM titanium [12]. In comparison to previously published data on tricalcium phosphate (TCP) scaffolds, which had the same dimensions, pore arrangement and pore size [20], the results of the present *in vitro* study demonstrate that, regardless of the manufacturing method, the biocompatibility of the titanium used was clearly lower. The titanium alloy Ti6Al4V, which was used as base material for the titanium constructs, could be the main reason for these results. In literature, this material is already widely discussed with regard to its toxic properties. In this context, it is assumed that vanadium can induce the release of reactive oxygen species (ROS), which adversely affect cell survival [22,23]. In the production process, the entire titanium surface is covered with a natural oxide coating to reduce the corrosion potential of the metal [24]. However, this coating can be destroyed by chemical substances or abrasion particles [25], which may lead to a release of ROS. Furthermore, cells that have come into contact with the titanium surface can produce larger quantities of ROS, such as H_2_O_2_. These ROS, in turn, react with the oxide coating, resulting in the production of additional free radicals. The cells are therefore exposed to permanent oxidative stress, which can have a negative effect on the viability and thus the survival of cells [25]. Due to the different biocompatibility of the titanium surfaces, the additive manufacturing methods can have a major influence on the survival of cells. At the macroscopic level, selective LBM produced mainly smooth surfaces and edges (Figure 1d). In contrast, the surfaces of the EBM scaffolds were significantly more uneven and showed many small particle inclusions, which could also be detected by scanning electronic microscopy (Figure 1g). These particle inclusions resulted from titanium powder residues that had not been melted down into the smooth titanium surface during the melting process. These particles could destroy the oxide coating of the titanium surface and accelerate corrosion processes. Moreover, we have demonstrated that abrasion particles from TiO_2_ can have a negative impact on cell viability [26]. The EBM titanium scaffolds thus seem to have less favorable material properties, which may adversely affect biocompatibility in the static cell culture. To actively eliminate possible adverse factors (e.g., ROS, particles) *in vitro*, the titanium scaffolds should be integrated into a dynamic cell culture system in further studies. 

Another important aspect in the evaluation of bone substitute materials is their influence on the differentiation potential of cells. On the one hand, the materials should constitute an artificial extracellular matrix and, on the other hand, they should support endogenous matrix synthesis by the cells cultivated on them. In the course of cultivation, an increase in the synthesis of collagen type 1 was demonstrated in both materials examined. Nevertheless, we tried to determine the concentrations between center and periphery, it should be noted that the verification is methodically limited. Therefore, an immunohistological staining could be performed to receive results. This should be established in further studies. However, an increase of osteoblast differentiation was detected on both titanium scaffolds.

### 4.2. Oxygen Supply and Acidification within the Bone Substitute Materials

Currently, the knowledge on optimal oxygen conditions in human bone tissue is insufficient, but it is known that the mean tissue oxygen levels are between 1% and 9% [27]. The data obtained in this study confirm that sufficient oxygen supply to human bone cells within the titanium constructs could be ensured over the test period. The oxygen content within the titanium scaffolds showed a smaller decrease between day 0 and 8 day (EBM titanium: −5.74% and LBM titanium: −1.3% in comparison with TCP: −7.55% [20]). In the EBM titanium, a significant difference between periphery and center was only observed after seven days of cultivation. In the LBM titanium, a significant difference was not observed until the end of the test. This divergent oxygen supply within the scaffolds is mainly due to their different colonization with bone cells. In the course of the test period, it was shown that the sintered TCP scaffolds [20] allowed significantly better seeding of human bone cells than the titanium scaffolds. Therefore, the oxygen consumption is increased due to an increase in the number of cells during the cultivation period. However, it can be concluded from the results that the evenly distributed macropores allow an adequate oxygen supply by diffusion in a static cell culture. 

Tissue hypoxia leads to local acidification, which is caused by the anaerobic cell metabolism and the lactic acid production associated with it. The exposure to hypoxia as well as reduced pH values can stimulate osteoclasts and their resorptive properties [28]. Microsensor-based pH monitoring revealed a slight acidification in the bone substitute materials investigated. Slight pH deviations can already inhibit the mineralization of organic matrix by osteogenic cells [28]. However, the tests performed within this study showed an increase in the synthesis of extracellular matrix in both bone substitute scaffolds. The acidification within the scaffolds thus revealed no side effect on the collagen synthesis. In addition, the diffusion of nutrients and oxygen can be reduced by increasing collagen deposition in the pores of the scaffolds [15], which favors hypoxia within the structures. Since no hypoxia occurred in the center of the titanium constructs, it can be assumed that cell seeding and the deposition of extracellular matrix did not reduce the size and regular arrangement of macropores to such an extent that diffusion processes in the static cell culture were measurably affected.

## 5. Conclusions 

In conclusion, the results of the present study demonstrate that monitoring of the oxygen concentration and cell viability in large-area bone substitute materials is an essential requirement for *in vitro* assessment of materials, pore design and pore size. However, further studies are required to verify the biomechanical and biological suitability of the bone substitute materials *in vivo* in an adequate animal model.
